# When Dread Risks Are More Dreadful than Continuous Risks: Comparing Cumulative Population Losses over Time

**DOI:** 10.1371/journal.pone.0066544

**Published:** 2013-06-26

**Authors:** Nicolai Bodemer, Azzurra Ruggeri, Mirta Galesic

**Affiliations:** 1 Center for Adaptive Behavior and Cognition, Max Planck Institute for Human Development, Berlin, Germany; 2 Harding Center for Risk Literacy, Max Planck Institute for Human Development, Berlin, Germany; Universitá del Piemonte Orientale, Italy

## Abstract

People show higher sensitivity to dread risks, rare events that kill many people at once, compared with continuous risks, relatively frequent events that kill many people over a longer period of time. The different reaction to dread risks is often considered a bias: If the continuous risk causes the same number of fatalities, it should not be perceived as less dreadful. We test the hypothesis that a dread risk may have a stronger negative impact on the cumulative population size over time in comparison with a continuous risk causing the same number of fatalities. This difference should be particularly strong when the risky event affects children and young adults who would have produced future offspring if they had survived longer. We conducted a series of simulations, with varying assumptions about population size, population growth, age group affected by risky event, and the underlying demographic model. Results show that dread risks affect the population more severely over time than continuous risks that cause the same number of fatalities, suggesting that fearing a dread risk more than a continuous risk is an ecologically rational strategy.

## Introduction

Imagine two different risky events: One threatens to kill 100 people at once; the other threatens to kill 10 people every year over a period of 10 years. The first event represents a *dread risk,* a rare event that kills many people at once, such as a pandemic, an earthquake, or a terrorist attack. The second event represents a *continuous risk*, a relatively frequent event that kills many people over a longer period of time, such as diabetes, air pollution, or car accidents. Which of the two risks is more severe? Both events kill the same number of people and differ only with respect to the time frame. Yet, people react much more strongly to dread risks than to continuous risks, in terms of both perception and avoidance behavior [1]–[3]. For instance, in reaction to the 9/11 terrorist attacks (a typical dread risk), many Americans avoided air travel and switched to their cars without considering that the risk of dying in a car accident (a continuous risk) is larger than the risk of an airplane terrorist attack, and even of dying in an airplane accident in general [4]. The avoidance of flying and the elevated use of cars increased the number of fatal highway crashes after the 9/11 attacks [1], [2], [5].

People’s higher sensitivity to dread risks compared with continuous risks is often considered a bias: If the continuous risk causes the same number of fatalities, it should not be perceived as less dreadful. In this paper we offer an alternative explanation to the assumption of biased minds and argue that a stronger reaction to dread risks is ecologically rational, because dread risks actually cause a larger cumulative reduction in the population size.

Different hypotheses have been proposed to explain why people fear dread risks more than continuous risks. First, the psychometric paradigm [3] suggests that high lack of control, high catastrophic potential, and severe consequences account for the increased risk perception and anxiety associated with dread risks. Second, people might lack knowledge about the statistical information underlying risks [6], in particular about the large number of fatalities caused by continuous risks. Third, because people estimate the frequency of a risk by recalling instances of its occurrence from their social circle or the media, they may overvalue relatively rare but dramatic risks and undervalue frequent, less dramatic risks [7], [8]. This is further supported by findings that people generally overestimate low probabilities and underestimate high probabilities [9]–[11], although the observed pattern can be partially explained with a regression-to-the-mean effect [8]. Fourth, according to the preparedness hypothesis, people are prone to fear events that have been particularly threatening to survival in human evolutionary history [12]. Given that in most of human evolutionary history people lived in relatively small groups, rarely exceeding 100 people [13], [14], a dread risk, which kills many people at once, could potentially wipe out one’s whole group. This would be a serious threat to individual fitness, as being in a group reduces predation risk, helps with finding food and hunting, and increases survival chances when injured [15], [16]. In line with this hypothesis, Galesic and Garcia-Retamero [17] found that people’s fear peaks for risks killing around 100 people and does not increase if larger groups are killed.

A different perspective reveals that dread risks lead to significantly worse short- and medium-term consequences than continuous risks, even if they do not eliminate a whole group. Thus, we focus not only on the overall number of immediate fatalities, as in previous accounts, but also on (a) the population size over time, and (b) the role of the age group that is affected by the risky event. Note that a fatal event strikes twice: it kills a number of people immediately, and it reduces the number of future offspring by reducing the number of their potential parents. A risk that affects children and young adults will have stronger negative effects on future group growth than a risk that affects group members who are past their reproductive period. Dread risks such as pandemics, terrorist attacks, or nuclear accidents are more likely to strike children and young adults compared to many continuous risks such as diabetes, cancer, heart attack, or household accidents, which affect primarily older people [18]. For example, the H1N1 pandemic in 2009 was more likely to infect younger people, whereas older people were relatively immune, probably due to previous exposure to a similar virus strain [19].

We hypothesize that dread risks cause larger cumulative losses on the population level than continuous risks. More specifically, we hypothesize that the number of people-years lost because of a dread risk is larger than the number of people-years lost because of a continuous risk, in particular when the event affects the younger age groups. People-years correspond to the number of people who live 1 year in the population. Hence, by killing a large number of children or young adults at once, dread risks not only deprive the society of their contribution in subsequent years, but they also remove the potential contribution of the offspring the victims could have had if they had survived longer.

To illustrate this hypothesis, consider first a very simplified example. Imagine a population of 40 people, uniformly distributed across four age groups:

*Children and adolescents, aged 0–19 years*: Pre-fertile generation that may produce offspring in the future.

*Young adults, aged 20–39 years*: Fertile generation that currently produces offspring.

*Older adults, aged 40–59 years*: Post-fertile generation.

*Elderly adults, aged 60–79 years*: Post-fertile generation.

**Figure 1:**
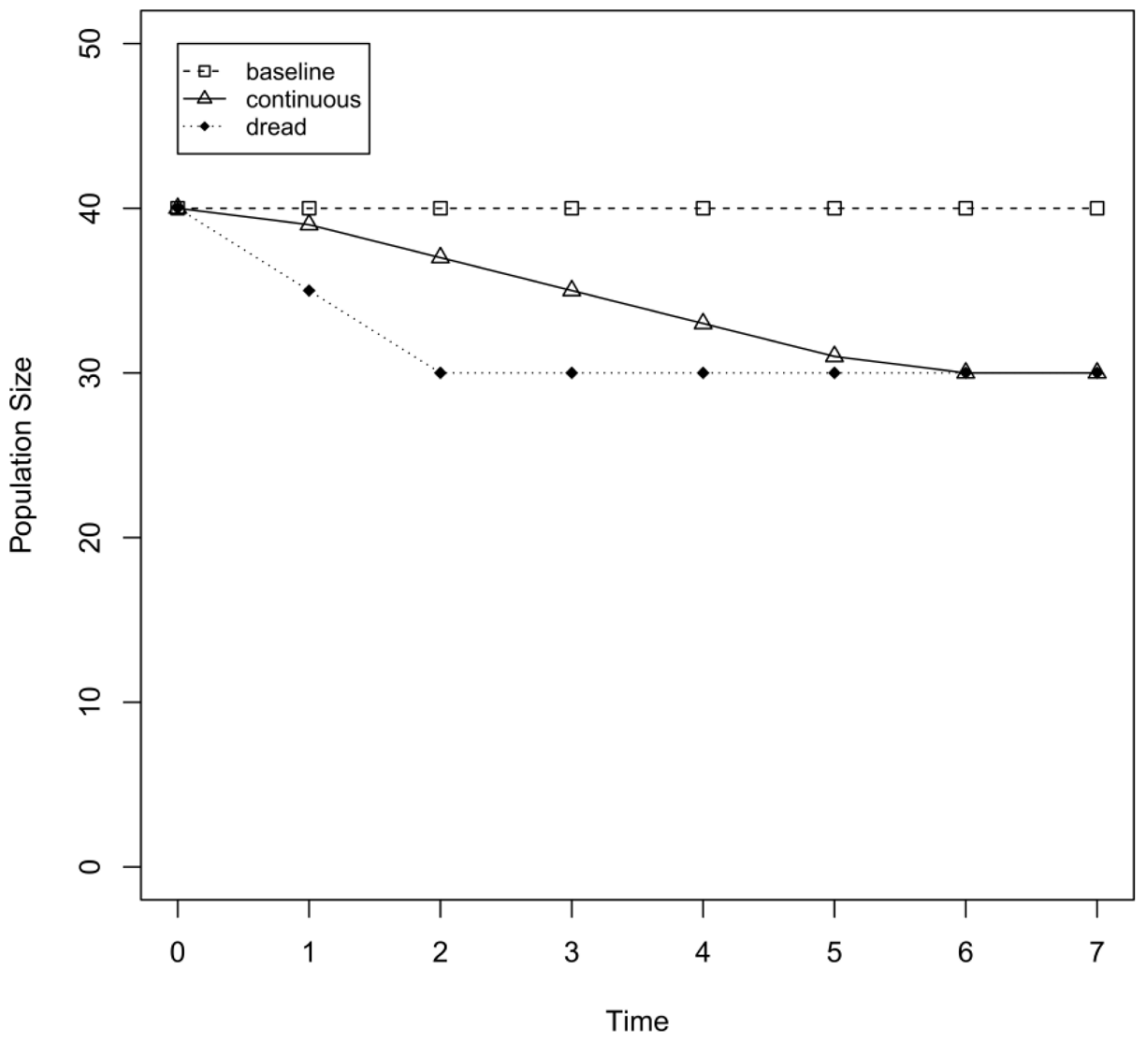
Impact of continuous and dread risk event on cumulative population size. Development of the population size when no risky event is present (baseline), and when a continuous risk (1 individual killed from *t*_1_ to *t*_5_) or a dread risk (5 individuals killed at *t*_1_) event occurs. A dread risk leads to a more immediate impact on cumulative population size that lasts longer compared with the continuous risk.

Further assume that the population growth is constant and that every year each young adult produces exactly one offspring. This implies that the number of children at time point *i*, *t_i_*, corresponds to the number of young adults at time point *i*-1, *t_i_*_-1_. Moreover, at every *t_i_* a generation shift takes place, so that the number of young adults at *t_i_*_+1_ corresponds to the number of children at *t_i_*, and so on for the other groups. Moreover, all elderly adults at *t_i_*_-1_ will be dead at *t_i_*. In the absence of any dread risk or continuous risk, the population is constant over time with *N*_total_ = 40 (see Figure 1).

What happens if a dread risk occurs at *t*_1_ that kills 50% of the young adults (i.e., 5 young adults)? At *t*_1_, the total population is reduced to *N*_total_ = 35 (*N*_children_ = 10, *N*_young adults_ = 5, *N*_older adults_ = 10, *N*_elderly adults_ = 10). At *t*_2_ the population is further reduced to *N*_total_ = 30 (*N*_children_ = 5, *N*_young adults_ = 10, *N*_older adults_ = 5, *N*_elderly adults_ = 10), because the number of newborn offspring is smaller due to the fewer young adults. Finally, the population size settles at *N*_total_ = 30, with continuous fluctuation within the respective groups.

What happens if a continuous risk, a disease, occurs at *t*_1_ that kills five young adults over a period of five time steps (one young adult at every *t*_i_, from *t*_1_ to *t*_5_)? Note that the total number of fatalities directly caused by the risk is the same as in the dread risk scenario (i.e., 5). The total population is reduced to *N*_total_ = 39 at *t*_1_ and continues to decline until *t*_6_, where it finally corresponds to the size of the population hit by the dread risk (see Figure 1).

In sum, the continuous risk takes five more generations to affect the population as severely as the dread risk. The difference in the cumulative losses caused in the population by the dread versus continuous risk, can be calculated by determining the area between the curves representing the difference in the cumulative population sizes of the two conditions (i.e., the difference in people-years over time). In the example in Figure 1, this integral is 20, meaning that the population hit by the dread risk lost 20 people-years more than the population experiencing the continuous risk.

This simple illustration shows that people’s tendency to fear dread risks more than continuous risks can be ecologically rational, because dread risks can affect the population more severely in the long run. It is important to note that we do not claim this to be necessarily a universal pattern. We do not exclude there might be situations (i.e., a set of parameters) in which a continuous risk causes stronger cumulative losses than a dread. However, this is the pattern that occurs in all our simulations. In the following, we present results of two sets of more fine-tuned simulations.

## Simulation Set 1

In the first set of simulations, we assumed a small population size, similar to groups in which people lived throughout most of evolutionary history [14]. We manipulated whether the population growth rates were constant, increasing, or decreasing, and which age group was exposed to a dread or to a continuous risk.

### Methods

We set the total population to 160 people. The individuals were distributed equally across 80 years (i.e., there were 2 individuals for each age at *t*_0_) and across four age groups, as in the illustrative example above. Between conditions, we manipulated (a) whether a dread or a continuous risk occurred, (b) the population growth rate, and (c) which age group was hit by the risk. The risk simulated was either a dread risk that immediately killed 50% of the population of the age group hit, or a continuous risk that killed the same total number of people in the same age group over a period of 10 years. The population growth rate was manipulated by setting the birth rate to either 0.05 (constant population), 0.075 (increasing population), or 0.025 (decreasing population). All individuals would die naturally after their 79^th^ year. The risk hit only children, only young adults, only older adults, or only elderly adults.

In total there were 24 scenarios. Each scenario was simulated 500 times, and we calculated for every time point the average population size within the simulations. We analyzed each scenario by comparing the log difference in cumulative people-years between the dread risk condition and the continuous risk condition after 25, 50, 75 and 100 years.

### Results

**Figure 2:**
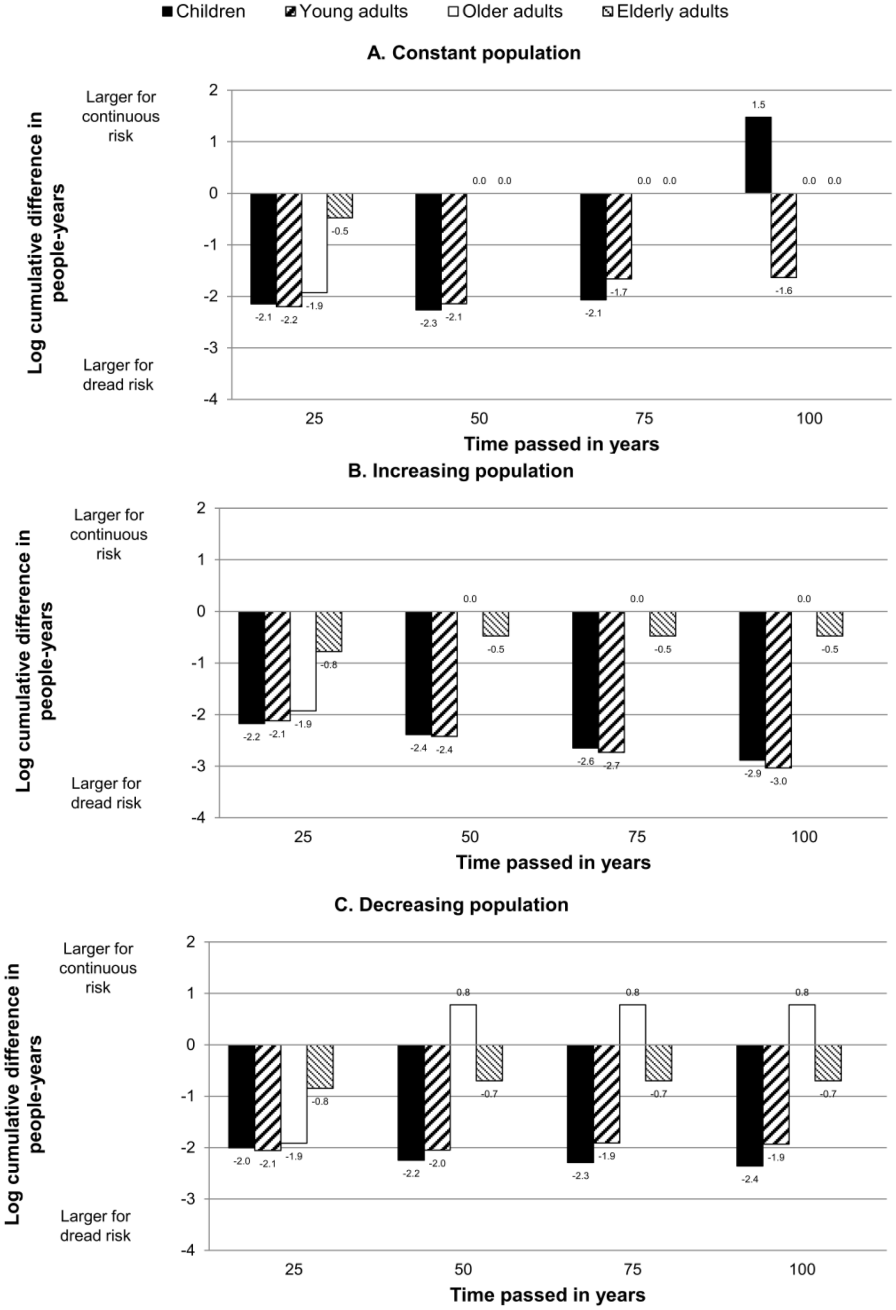
Log difference in people years lost after continuous and dread risk event (population size: 160). Log difference in people-years lost because of continuous and dread risk, by age group hit by the risk, separately for A. constant, B. increasing and C. decreasing populations. The dread risk killed 50% of a specific age group at once; the continuous risk the same total number of people over a period of ten years. A negative value of the difference indicates that the loss in people-years is larger for the dread risk; a positive value that the loss is larger for the continuous risk. Results show that dread risks lead to larger losses in people-years across time compared with continuous risks, in particular when children and young adults are affected.

Figure 2 shows the results for the log difference in cumulative people-years depending on the population growth rate and the hit group after 25, 50, 75, and 100 years. A zero value indicates no difference in cumulative people-years between the dread risk and continuous risk; a negative value indicates a higher loss in cumulative people-years in the dread risk condition, and a positive value a higher loss in the continuous risk condition.

When children and young adults were hit by the risks, the effect was stronger and lasted for the entire 100-year-range simulated. When older and elderly adults were hit, the difference between dread and continuous risks was weaker, decreased over time, and sometimes even became positive.

In sum, the results show that the dread risk affected the cumulative population size more strongly for most scenarios, particularly when it hit children or younger adults. The objective of this first set of simulations was to evaluate the impact of a dread and a continuous risk on small samples that would reflect the sample size of social circles. With a second set of simulations we investigated the effects of such risks on a much larger population of the size of the U.S. population in 2010.

## Simulation Set 2

### Methods

We set the population size to the actual U.S. population size in 2010 [20] with the respective age distributions and population growth rates. Because the statistics only provided population size for age groups–for instance, 20,201,362 children <5 years old lived in the United States in 2010–we assumed an equal distribution of the children across 0–4 years for reasons of simplicity. As in Simulation Set 1, we manipulated which age group (children, young adults, older adults, elderly adults) was hit by the risk. The risk killed either 20% of the hit group, or the same total number of people over 10 years.

We again ran 500 simulations for each scenario, calculated the averaged population size of the dread risk and continuous risk and plotted the log integrals after 25, 50, 75, and 100 years.

### Results

**Figure 3:**
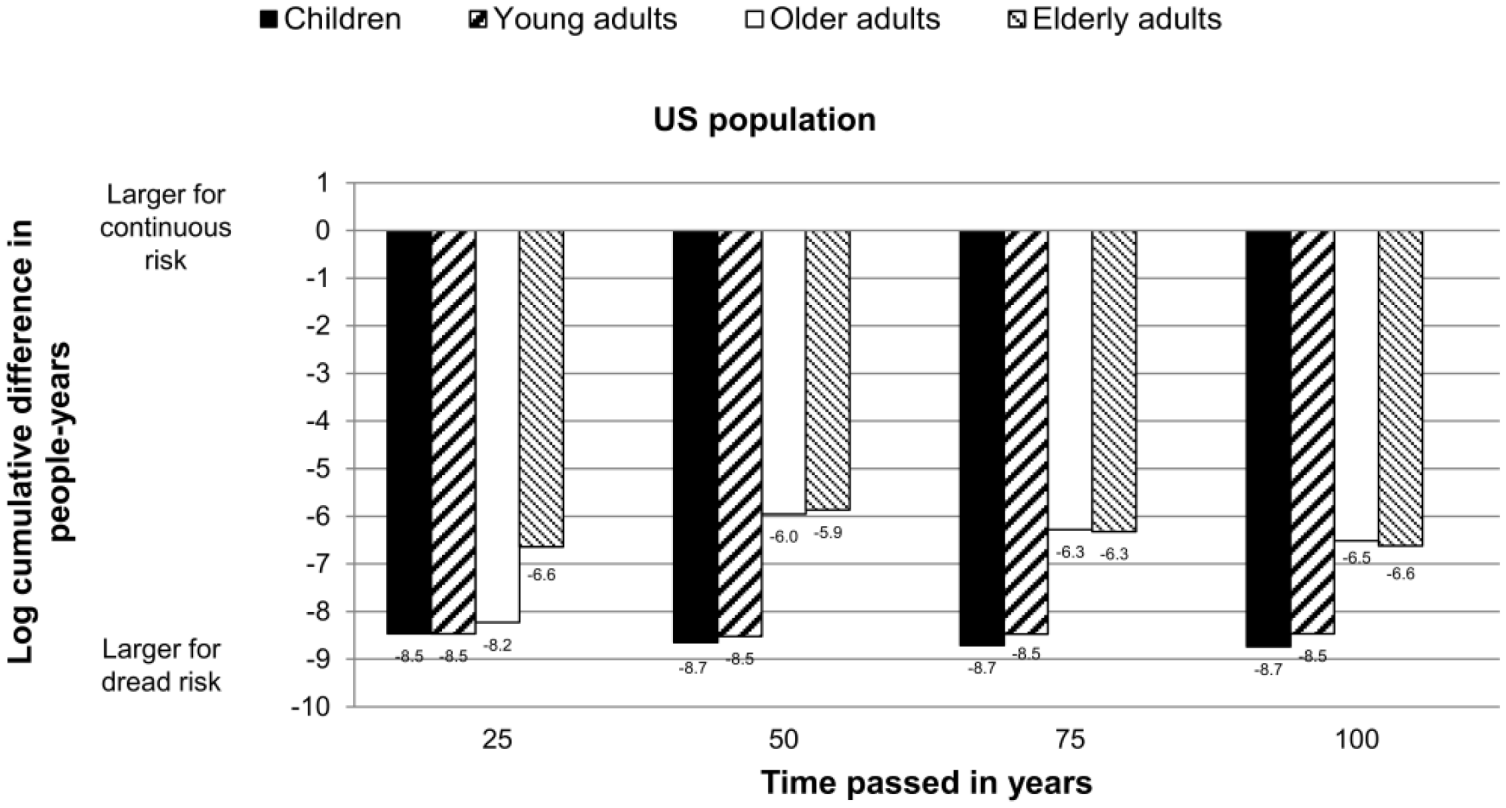
Log difference in people years lost after continuous and dread risk event (US population). Log difference in people-years lost because of continuous and dread risk, based on the US population. The dread risk killed 20% of a specific age group at once; the continuous risk killed the same total number of people over a period of 10 years. Results show that the dread risk leads to a larger loss in people-years over time across all age groups. The loss was largest when children and young adults were affected.

Using real U.S. data, we found support for the findings of the previous simulations. The differences between the cumulative population hit by dread versus continuous risks occurred across all conditions and lasted over, at least, 100 years. Independent of which age group was affected, the dread risk led to a higher loss in people-years than the continuous risk (Figure 3). Loss was highest when children and young adults were hit by the risk.

## Simulation Set 3

The third set of simulations wanted to test whether the results obtained from the first two sets of simulations also hold when an alternative model underlies computation. We used ecology models to define population growth and the impact of a dread and continuous risk. Specifically, we used models described in Lande [21] and Barnthouse [22], who investigated recovery rates of populations after catastrophes. Unlike in simulation sets 1 and 2, here we did not differentiate between the different age groups affected by the dread and continuous risk. Instead, the risky event simply reduced an a priori specified proportion of the population.

### Methods

We used a basic growth model of population, given the current population size *N_t_*, the growth rate *r* and the carrying capacity *K* (i.e., the carrying capacity represents the maximum population size possible, see [22]):
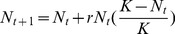
(1)


We specified that a dread risk kills a particular proportion of the population (see also [21]):
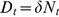
(2)


Immediately after the dread, the remaining population grows as follows:
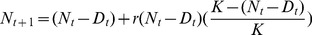
(3)


In the following time steps, the population again grows according to (1). For the continuous risk, the same total amount of people as in the dread was killed, but over a period of *x* years:
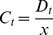
(4)


During these *x* years, the remaining population grows as follows:
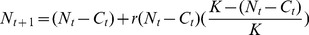
(5)


We use these formulas to simulate changes of population size subject to dread and continuous risks. An analytic solution for time to extinction of a population after random catastrophes is given in Lande [21].

In our simulations, we set the growth rate to either *r* = .01 (increasing population), *r* = 0 (constant population), *r* = -.01 (decreasing population). The initial population size at *t*_0_ was set to 100 people and the carrying capacity to *K* = 10,000. The dread risk eliminated *δ* = .3 of the population, the continuous risk eliminated the same total number of people over a period of 10 years. We followed the change of population size over a period of 100 years. The two risks could occur at any time within such time frame, but always occurred simultaneously, that is, they hit the population at the same *t*. Note that in simulation 1 and simulation 2 the risky events always occurred at the beginning of the time period. We simulated every condition 10,000 times and calculated the average integral, signaling the difference in cumulative people-years after the entire period of 100 years.

## Results

In line with the findings of the previous simulations, we found that, over the time period considered, the dread risk resulted in higher losses in cumulative people-years than the continuous risk. The integral for the increasing population was −906, for the constant population −355, and for the decreasing population −137. Hence, even when using another model to test the impact of dread and continuous risks and even when not differentiating between different age groups, the results of the first two sets of simulations still hold.

## Discussion

People’s stronger reaction to dread risks compared with continuous risks is often perceived as a bias. This result proposes a new perspective against which the current hypotheses accounting for people’s perception and reaction to dread risks might be reconsidered.

We showed through three different sets of simulations that this is in fact an ecologically rational strategy. The effect of dread risks compared with continuous risks is amplified twice: First by killing more people at a specific point in time, and second by reducing the number of children and young adults who would have potentially produced offspring. Hence, this effect is particularly strong when children and young adults are hit which is often the case for dread risks (e.g., earthquakes, terrorist attacks, pandemics). This result is also in line with findings suggesting that people are more concerned about risks killing younger, and hence more fertile, groups [23]. Moreover, when using a population ecology model and without specifying the age group affected by the respective risky events, the conclusion still holds.

Where does the fear of dread risk come from? Although our study was not designed to provide this answer, we can speculate about some possible answers to this question. According to the preparedness hypothesis mentioned at the beginning, people may be prone to fear risks that threaten their whole group. This trait may be a product of either individual or group selection. Because individual fitness depends and improves with group living [24], in particular in conditions of scarce population density that prevailed throughout much of human evolutionary history [25], an individual might profit from developing alertness to events that threaten to kill her group. An argument could also be made for group selection [26]. Groups that were more alert to dread risks and therefore managed to avoid them, suffered less from dread risks’ devastating long-term consequences, which in turn would make them less vulnerable to other groups. Besides the evolutionary arguments, it is also possible that people learn to fear and avoid risks that appear to be particularly dangerous in their current environment. However, at this point we can only speculate about these explanations and further studies could be designed to address them.

There are important practical implications of this finding. For instance, from a public policy perspective, an appropriate reaction to dread risks would be to stimulate increase in birth rates and/or immigration to counterbalance the stronger loss in population size.

In sum, people’s fear and stronger risk perception of dread risk, compared to continuous risks, should not be considered an irrational bias, an emotional overreaction to a dramatic event. In fact, people’s intuition seems to capture the objective severity of the two different risks.
